# Group benefit associated with polymorphic trichromacy in a Malagasy primate (*Propithecus verreauxi*)

**DOI:** 10.1038/srep38418

**Published:** 2016-12-02

**Authors:** Carrie C. Veilleux, Clara J. Scarry, Anthony Di Fiore, E. Christopher Kirk, Deborah A. Bolnick, Rebecca J. Lewis

**Affiliations:** 1 Department of Anthropology, University of Texas at Austin, 2201 Speedway Stop C3200, Austin, TX, 78712, USA; 2 Population Research Center, University of Texas at Austin, Austin, TX, 78712, USA.

## Abstract

In some primate lineages, polymorphisms in the X-linked *M*/*LWS* opsin gene have produced intraspecific variation in color vision. In these species, heterozygous females exhibit trichromacy, while males and homozygous females exhibit dichromacy. The evolutionary persistence of these polymorphisms suggests that balancing selection maintains color vision variation, possibly through a ‘trichromat advantage’ in detecting yellow/orange/red foods against foliage. We identified genetic evidence of polymorphic trichromacy in a population of Verreaux’s sifaka (*Propithecus verreauxi*) at Kirindy Mitea National Park in Madagascar, and explored effects of color vision on reproductive success and feeding behavior using nine years of morphological, demographic, and feeding data. We found that trichromats and dichromats residing in social groups with trichromats exhibit higher body mass indices than individuals in dichromat-only groups. Additionally, individuals in a trichromat social group devoted significantly more time to fruit feeding and had longer fruit feeding bouts than individuals in dichromat-only groups. We hypothesize that, due to small, cohesive sifaka social groups, a trichromat advantage in detecting productive fruit patches during the energetically stressful dry season also benefits dichromats in a trichromat’s group. Our results offer the first support for the ‘mutual benefit of association’ hypothesis regarding the maintenance of polymorphic trichromacy in primates.

Researchers have long noted that primates are unique among placental mammals in exhibiting trichromatic color vision^1^,^2^. Whereas most other mammals are dichromats and can distinguish color along a single dimension (shorter wavelengths *vs*. longer wavelengths, or blues/violets *vs*. yellows/greens/reds), trichromats can distinguish not only between these shorter and longer wavelengths but also between middle and long wavelengths (i.e., greens *vs*. reds: Fig. 1). Anatomically, trichromacy in primates is achieved through the presence of three types of retinal cone photoreceptors that are maximally responsive to either short wavelengths (S cones), middle wavelengths (M cones), or long wavelengths (L cones); however, the genetic mechanisms underlying trichromacy differ between major primate clades^2^. All catarrhine primates (Old World monkeys, apes, humans) exhibit routine trichromacy due to a fixed duplication of the *M*/*LWS* opsin gene that allows normal individuals to produce both M and L retinal cones^3^,^4^. In contrast, most platyrrhines (New World monkeys) and some lemurs exhibit polymorphic trichromacy, wherein different alleles encoding M or L cones are found at the X-linked *M*/*LWS* opsin locus^5^,^6^,^7^,^8^. In these species, females that are heterozygous at the *M*/*LWS* locus develop separate M and L retinal cones, which –in conjunction with the autosomally-encoded S cone— produce trichromacy. Meanwhile homozygous females and all males develop a single type of M/L cone and thus have dichromatic color vision^2^.

Since sex-linked polymorphic trichromacy was first identified, researchers have sought to understand the ecological factors influencing its evolution and maintenance^7^,^9^,^10^,^11^,^12^,^13^. The long-term persistence of polymorphic trichromacy suggests that balancing selection acts to maintain color vision diversity in some primate species^7^,^9^,^14^. However, substantial debate exists^7^,^9^,^12^,^13^,^14^,^15^ regarding which form of balancing selection is acting on the *M*/*LWS* locus to maintain polymorphic trichromacy (i.e., ‘heterozygote advantage’, ‘niche divergence’, or ‘mutual benefit of association’).

Under the heterozygote advantage hypothesis, trichromacy is posited to improve detection of yellow/orange/red items (e.g., young leaves, fruit) against a green and brown foliage background^10^,^12^,^16^,^17^. Thus, trichromat females may experience a consistent foraging advantage (i.e., heterozygote advantage) relative to homozygous females. Increased reproductive output by trichromat females, due to shorter interbirth intervals or increased offspring survival associated with higher energy gain, would act to maintain the polymorphisms in the population^7^,^13^. However, because dichromatic color vision may be more effective than trichromacy in edge detection and breaking camouflage^18^,^19^, the possibility exists that dichromats have an advantage in some ecological contexts. Consequently, the ‘niche divergence’ hypothesis posits that the *M*/*LWS* opsin polymorphism could be maintained through frequency dependent selection, wherein the fitness of a color vision phenotype is related to its relative frequency in the population^7^,^13^. Trichromats and dichromats may thus be able to achieve similar reproductive fitness by exploiting distinct ecological niches (e.g., foraging for ripe fruit *vs*. cryptic insects)^13^,^15^. Finally, as an alternative to either of these scenarios, the ‘mutual benefit of association’ hypothesis suggests that individuals of both phenotypes may gain fitness benefits by residing in a social group with members of a different phenotype^9^,^13^,^14^. For example, trichromats may benefit from the earlier detection of predators by dichromats, while dichromats may experience reduced foraging costs through associations with trichromats who are able to detect higher quality resource patches at greater distances^20^.

To date, most efforts have focused on the heterozygote advantage hypothesis, seeking to identify a clear foraging advantage to trichromacy. Experimental studies with captive primates have confirmed that, in some species, trichromats are more efficient than dichromat conspecifics at finding yellow/orange/red targets^21^ and simulated ripe fruit^22^. Physiological modeling studies similarly suggest that trichromats should be better at detecting yellow/orange/red fruits in natural habitats^12^,^16^. Nevertheless, field researchers have struggled to detect clear evidence of a trichromat advantage in natural foraging tasks. In platyrrhine species, trichromats and dichromats do not appear to differ in food intake or energy acquisition rates^23^, in successfully finding food patches^24^,^25^, in the proportion of time spent feeding on fruit or insects^15^,^26^, or in short range fruit foraging efficiency^27^. While trichromat capuchins (*Cebus capucinus*) may be able to better detect ripe figs, feeding intake rates do not differ among color vision phenotypes, implying that dichromats achieve similar total intake using non-color-based cues^28^. Consistent with this finding, long-term demographic data for capuchins reveal no difference between trichromats and dichromats for any measure of reproductive success^29^.

In the absence of a consistent trichromat advantage, researchers have begun exploring alternate hypotheses, such as the niche divergence and mutual benefit of association hypotheses. For example, in support of the niche divergence hypothesis, studies of humans and captive non-human primates have found that dichromats are more efficient than trichromats at finding camouflaged food items, particularly in shaded light environments^18^,^19^,^30^. Field studies of capuchins similarly suggest that dichromats are more efficient at capturing camouflaged exposed insect prey and spend less time visually foraging for insects than trichromats, while trichromats are more efficient at extracting embedded insects^15^,^31^,^32^. However, these differences in foraging efficiency do not extend to cryptic and conspicuous fruit^31^. Furthermore, no effect of color vision phenotype on insect capture attempts and successes has been found in wild squirrel monkeys^26^. Unlike the heterozygote advantage and niche divergence hypotheses, the mutual benefit of association hypothesis has not yet been explicitly tested in any primate population^13^,^14^. Based on previous studies, researchers have hypothesized that trichromacy may be especially advantageous for long-distance detection of fruit or young leaves^10^,^12^,^17^. Consequently, some researchers have proposed that trichromacy may benefit group members through visual discovery of more productive food patches at greater distances, particularly in species with small social groups or with subgrouping where all individuals would be able to exploit the patch^26^,^33^.

While polymorphic trichromacy has been identified in at least three diurnal and cathemeral lemur genera (Lemuridae: *Eulemur*, *Varecia*, Indriidae: *Propithecus*)^5^,^6^,^8^, most field studies to date have focused on platyrrhines. Very little work has examined the ecology of color vision in trichromat or potentially trichromat lemur populations^34^,^35^,^36^. Although trichromacy among lemurs may be functionally different from trichromacy in platyrrhines due to lower retinal cone density^37^ and lower visual acuity^38^,^39^, experimental evidence suggests that trichromacy can influence lemur foraging behavior^40^.

In this study, we used *M*/*LWS* opsin genotyping to document the existence of polymorphic trichromacy in a wild lemur population and conducted the first explicit test of fitness effects of that trichromacy. Specifically, we explored the relationships between color vision phenotype, proxy measures of reproductive success, and feeding behavior in a wild population of Verreaux’s sifaka (*Propithecus verreauxi*), a ~3 kg diurnal indriid. The study was conducted at the Ankoatsifaka Research Station in the dry deciduous forest of Kirindy Mitea National Park, western Madagascar. Sifaka are primarily folivorous and live in small cohesive social groups of 1–3 adult females and 1–3 adult males (2–13 total individuals)^41^,^42^,^43^,^44^. Females are dominant to males^45^ and more likely to lead group movement^46^, providing opportunities for trichromat females to lead their small groups to more productive food patches. Sifaka thus offer an excellent system in which to explicitly test the mutual benefit of association hypothesis.

We hypothesized that trichromacy is advantageous at an individual level, leading to higher fitness in trichromat females relative to dichromat females. Additionally, if trichromacy provides a group-level selective advantage, we expect both trichromats and dichromats co-residing in social groups with trichromats to have higher fitness than individuals in dichromat-only social groups. To test these hypotheses, we first surveyed the *M*/*LWS* opsin gene in the sifaka population at Kirindy Mitea National Park to identify color vision phenotypes. Then, using nine years of morphological and demographic data, we tested whether individual color vision phenotype or membership in a polymorphic trichromat group influences three potential fitness proxies: body mass index during the dry season, reproductive output, and infant survival. We predicted that trichromats or dichromats residing with trichromats show higher body mass indices, female reproductive output, and infant survival to the first year. Finally, using a large existing dataset of feeding behavior for four groups, we explored whether an individual’s color vision phenotype or membership in a trichromat social group influences feeding time budget, intake rate, or length of time in a feeding tree for food items where color is likely to be an important cue for detection (i.e., fruit or young leaves). If trichromacy provides a benefit to the entire social group, dichromat individuals residing with a trichromat should have higher values for some or all of these measures of foraging success compared to dichromats residing in dichromat-only groups.

## Results and Discussion

### *M*/*LWS* Opsin Genotyping and Prevalence of Trichromacy in a Wild Sifaka Population

We genotyped the *M*/*LWS* opsin gene in 55 sifaka across nine social groups around the Ankoatsifaka Research Station at Kirindy Mitea. Of the 31 females tested, 7 were heterozygous (22.6%) for the M-sensitive 543 nm allele and the L-sensitive 558 nm allele, and thus genetically trichromat. These trichromats were members of four social groups. The remaining females (*n* = 24, 77.4%) were homozygous for the 558 nm allele and genetically dichromat. Among the dichromat males, 7 possessed the 543 nm allele (29%) and 17 possessed the 558 nm allele (71%). Thus, the 558 nm allele occurred at a much higher frequency (83.7% of chromosomes) than the 543 nm allele.

Although trichromacy has been identified in captive Coquerel’s sifaka^5^,^6^,^40^ (*Propithecus coquereli*), this study is the first published evidence of the *M*/*LWS* opsin gene polymorphism in Verreaux’s sifaka (*Propithecus verreauxi*), as well as one of the first estimates of *M*/*LWS* allele frequencies in a wild lemur population. While the frequency of trichromacy ranges from 35 to ~60% in platyrrhine populations^14^,^23^,^26^,^32^, it is lower in Verreaux’s sifaka, at less than one quarter of females. Our results also indicate that the longer wavelength allele (558 nm) is more common in this population, which is similar to a trend seen in some platyrrhine populations^12^,^26^. In platyrrhines, modeling studies found that dichromat genotypes can differ in their discrimination of stimuli, suggesting that allele frequencies may reflect selective pressures^12^,^47^. Interestingly, two other recent studies of the *M*/*LWS* opsin gene in wild lemur populations found that different alleles have become fixed in congeneric species of *Eulemur*; brown lemurs (*Eulemur fulvus*) uniformly possess the 543 nm allele^35^ while red-bellied lemurs (*E*. *rubriventer*) uniformly possess the 558 nm allele^36^. As more data on *M*/*LWS* opsin gene variation in wild lemur populations become available, it will be interesting to see how populations and species differ in both the frequency of trichromacy and allele frequencies.

### Effects of Color Vision Phenotype on Correlates of Reproductive Success

We investigated the effects of both individual color vision phenotype and group color vision phenotype on three presumed correlates of reproductive success in Verreaux’s sifaka at Kirindy Mitea: body mass index (BMI) during the dry season, reproductive output, and infant survival to the first year. The dry deciduous forest of western Madagascar is characterized by strong seasonality in rainfall and food availability, and sifaka typically experience a 10–20% loss of body mass in the dry season (May-Nov) relative to the short wet season (Jan-Mar)^42^,^43^. Because infants are born in July/August and weaned during the wet season^42^, the dry season also corresponds to periods of increased energetic demands on females due to reproduction (e.g., pregnancy, lactation)^42^,^48^.

We fitted linear mixed-effects models (LMMs) to test the effect of color vision phenotypes on 87 BMI measurements from 24 males and 13 females in seven social groups (four trichromat groups, three dichromat-only groups). Of these 37 individuals, 13 males, 2 dichromat females, and 5 trichromats resided in trichromat social groups for some duration between 2006 and 2015 (Supplemental Table 1). In a likelihood ratio test (LRT), the model including group color vision phenotype, pregnancy status, and sex to explain variation in BMI fit the data significantly better than a null model of sex and pregnancy status alone (χ^2^_1_ = 5.23, *p* = 0.022), suggesting that group color vision phenotype is an important influence on variation in sifaka BMI. Group phenotype (*p* = 0.029) was a significant factor in the model, but sex (*p* = 0.713) and pregnancy status (*p* = 0.369) were not. A second model treating color vision phenotype as a three-level factor (‘dichromat in dichromat-only social group’, ‘dichromat in trichromat social group’, ‘trichromat’) also fit the data significantly better than the null model of sex and pregnancy alone (LRT: χ^2^_2_ = 6.70, *p* = 0.035), which suggests that the ‘trichromat advantage’ in BMI extends to group mates, rather than being driven solely by an advantage to trichromat females. According to this three-level phenotype model, relative to dichromats in dichromat-only groups, trichromats had significantly higher BMIs (*p* = 0.046) while their dichromat group mates exhibited a trend (*p* = 0.085) for higher BMIs (0.95 and 0.85 higher BMI, respectively; Fig. 2). These higher BMIs among individuals in trichromat groups reflect a ~5% increase compared to dichromats in dichromat-only groups, and may confer a fitness advantage given the energetic stresses of the dry season, particularly those related to female reproduction^25^.

We next tested the effects of individual and group color vision phenotype on two binary reproductive measures: reproductive output and infant survival to the first year^49^. Previous studies of Verreaux’s sifaka at other sites^42^,^50^ found that females with higher body mass during the mating (wet) season were more likely to give birth and successfully rear an infant to one year. Given the trichromat individual and group advantage seen in dry season BMI, we predicted that trichromat females and dichromat females living in trichromat groups would exhibit higher reproductive success. Between 2006 and 2014, 59 infants were born into six social groups (three trichromat groups, three dichromat-only groups, 14 total mothers), 15 to trichromats (4 mothers) and 10 to dichromat members of trichromat groups (3 mothers). In contrast to our predictions, we found no significant effect of individual phenotype (LRT: χ^2^_1_ = 1.48, *p* = 0.224) or group phenotype (LRT: χ^2^_1_ = 0.35, *p* = 0.554) on whether a female gave birth in a given year (assessed over 79 reproductive cycles). Group color vision phenotype also did not affect infant survival to one year (LRT: χ^2^_1_ = 0.75, *p* = 0.385). However, we found a trend (LRT: χ^2^_1_ = 3.11, *p* = 0.078) suggestive of an increased likelihood of survival among infants born to trichromat mothers than infants born to dichromats (P(trichromat) = 0.832 vs. P(dichromat) = 0.679), consistent with our predictions based on dry season body condition.

Although the reproductive results were mixed, our findings offer the first evidence in primates that members of a social group including a trichromat female may experience a selective advantage. The dry season represents a period of major energetic stress for Verreaux’s sifaka, particularly for females^42^,^43^,^48^. Our results suggest that individual trichromacy and membership in a trichromat social group both may moderate some of the 10–20% loss in body mass^42^ that typically occurs during this period. Despite a small sample of mothers (*n* = 14 mothers, 59 infants), we detected a trend for infants born to trichromat females to have a greater probability of surviving the first year consistent with our predictions. Because any effects of color vision phenotype (either individual or group) on measures of reproductive success are likely small and relatively subtle^13^, this lack of significant results in our other reproductive analyses is not necessarily surprising, considering our small sample size and limited sampling period. Nevertheless, even modest benefits in reproductive success may be selectively important. For example, dominant female baboons give birth, on average, to only one additional offspring compared to subordinate females over the course of their reproductive lifespan^51^.

### Effects of Color Vision Phenotype on Sifaka Feeding Behavior

Given a group-level trichromat advantage in dry season body condition and a trend for increased infant survival among trichromat mothers, we next used an extensive existing dataset on individual feeding behavior collected over eight years to investigate potential mechanisms that might be responsible for these advantages. While BMI data and infant survival data included seven and six social groups, respectively (evenly represented by trichromat and dichromat-only groups), data on feeding behavior were only available for the four social groups that have been the focus of long-term data collection. Only one of these four groups contained trichromat females (Group II), and the two trichromats were the only adult females in the group. For these four social groups, average group size and home range size did not vary substantially between trichromat and dichromat-only groups over the study years^44^. Similarly, total observation hours did not substantially vary across the four groups (Supplemental Table 2).

Following previous studies of platyrrhine trichromacy^15^,^26^, we first investigated whether trichromats or members of the trichromat group spent more of their observation time budgets feeding on potentially color-relevant food types (e.g., fruit^16^, young leaves^17^) than dichromats or members of the three dichromat-only groups. Across groups, the average total time spent feeding did not substantially differ between wet (36%, standard deviation ±18%, *n* = 100 focal months) and dry (39 ± 12%, *n* = 306 focal months) seasons. However, consistent with the well-documented seasonal variation in food availability in sifaka habitats, the proportion of observation time spent feeding on fruit and young leaves varied between wet and dry seasons across all groups. In the wet season, sifaka devoted an average of 12% (±17%) of observation time feeding on fruit and 13% (±10%) on young leaves. By contrast, during the dry season, they spent 3% (±5%) of observation time feeding on fruit and 6% (±9%) on young leaves.

We fitted LMMs on logit-transformed proportional data of (1) time spent feeding, (2) time spent feeding on young leaves, and (3) time spent feeding on fruit relative to total observation time using fixed factors of color vision phenotype, sex, and season. After adjusting for multiple comparisons, neither individual color vision phenotype (LRT: χ^2^_1_ = 1.64, *p*_*adjusted*_ = 0.335) nor group phenotype (LRT: χ^2^_1_ = 0.18, *p*_*adjusted*_ = 0.835) significantly influenced total time spent feeding compared to a null model excluding color vision factors (*n* = 406 focal months, Supplementary Table 3). Consistent with the pattern of female dominance and feeding priority^50^, males spent less total time feeding than females (LRT: χ^2^_1_ = 9.82, *p*_*adjusted*_ = 0.017). Neither individual nor group color vision phenotype were significant factors in explaining the proportion of time spent feeding on young leaves (*n* = 321 focal months; individual LRT: χ^2^_1_ = 0.18, *p*_*adjusted*_ = 0.835; group LRT: χ^2^_1_ = 0.04, *p*_*adjusted*_ = 0.835). However, group phenotype (LRT: χ^2^_1_ = 8.62, *p*_*adjusted*_ = 0.017) was a significant positive factor in the proportion of time spent feeding on fruit, and the effect of individual phenotype (LRT: χ^2^_1_ = 5.27, *p*_*adjusted*_ = 0.054) approached significance after adjusting for multiple comparisons (*n* = 242 focal months, Table 1). To parse out the separate contributions of individual phenotype and group membership on the significant group phenotype effect, we performed a *post-hoc* LMM using the three-level color vision factor (Table 1). This three-level model also fit the data significantly better than the null model of sex and season (LRT: χ^2^_1_ = 8.67, *p*_*adjusted*_ = 0.044), indicating that trichromat individuals spent significantly more of their time budgets on fruit feeding (*p* = 0.018), whereas trichromat group mates showed a trend for more of their time budgets on fruit feeding (*p* = 0.068). This three-level model did not fit the data better than the individual phenotype (LRT: χ^2^_1_ = 3.35, *p*_*adjusted*_ = 0.131), nor the group phenotype model (LRT: χ^2^_1_ = 0.04, *p*_*adjusted*_ = 0.834), and suggests that we are potentially identifying both an individual and group-level effect of trichromacy.

Given this potential trichromat group advantage in fruit feeding, we further tested whether intake rates while feeding on fruit varied with color vision phenotype, similar to previous investigations in New World monkeys^23^,^27^,^28^. Using a subset of the behavioral data with recorded intake rates (measured in bites/minute), we fitted generalized LMMs (GLMMs) on fruit intake rate (*n* = 268 feeding bouts) with color vision phenotype, sex, and season as fixed effects (Supplementary Table 3). LRTs found that neither models including individual color vision phenotype (χ^2^_1_ = 0.72, *p* = 0.397) nor those including group color vision phenotype (χ^2^_1_ = 0.77, *p* = 0.379) fit the data significantly better than null models excluding color vision, suggesting that intake rate does not vary between the trichromat and dichromat-only groups. Consequently, the increased time spent by members of the trichromat group likely reflects greater total fruit intake on days where fruit is consumed, and thus potentially greater caloric intake.

Finally, we explored whether the trichromat group may be finding more productive fruit patches than the dichromat groups, which is one of the predictions of the mutual benefit of association hypothesis^20^,^26^,^33^. As a proxy of patch productivity, we calculated the length of time each focal individual spent actively feeding on fruit in a single tree as the sum of all consecutive feeding bouts in that tree. Because this value measures the length of time that an individual was actually feeding (e.g., chewing or processing food) and excludes search time, it may reflect the relative availability of fruit in a particular patch. During the wet season (*n* = 501 tree feeding bouts, Table 2), neither individual color vision phenotype (LRT: χ^2^_1_ = 2.47, *p* = 0.116) nor group color vision phenotype (LRT: χ^2^_1_ = 0.325, *p* = 0.568) had a significant effect on the length of time an individual fed in a single fruit tree. By contrast, in the dry season (*n* = 440 feeding bouts, Table 2), we found a significant effect of group color vision phenotype on the length of time an individual fed in a single fruit tree (LRT: χ^2^_1_ = 5.29, *p* = 0.021), but not individual phenotype (LRT: χ^2^_1_ = 0.79, *p* = 0.375). These results suggest that during the wet season when fruit is plentiful, trichromat groups do not have an advantage for finding more productive fruit patches. However, during the dry season, when fruit is much scarcer and fruit feeding occupies only ~3% of observation time (~13 minutes), members of the trichromat group spend an extra 1.2 min on average in each fruit patch. This additional time represents a ~27% increase in time spent feeding per tree compared to members of the dichromat groups.

One major drawback of our feeding data is that the sample included a single trichromat group; thus, we cannot be certain that differences in habitat quality, rather than trichromacy itself, are responsible for the trichromat group fruit feeding advantage. We cannot directly compare home range quality between the groups because phenological data are not available; preliminary analysis of habitat structure, however, suggests that trichromat and dichromat group ranges do not differ in stem density or stem height (Supplementary Fig. 1) or crown volume (Lewis, unpublished data). We extracted a proxy measure of habitat quality from the existing feeding data by comparing the number of individually-tagged plants used by each group for each of the seventeen species that sifaka use for fruit. In the dry season, the trichromat group used more individual plants for fruit (166 *vs*. 141, 136, and 126 for the three dichromat-only groups). However, when we examined the number of individual plants used for all parts over the year (e.g., young leaves, mature leaves, etc.), the trichromat group used fewer total individual plants of fruiting species than two of the dichromat-only groups (trichromat: 1047 trees; dichromat-only: 1191, 1186, and 833 trees).

Looking more closely at the differences among groups by plant species, the trichromat group utilized more individual plants for any plant part for only four species (possibly suggestive of greater availability of these particular species in the trichromat group’s range). For only two of those species did the trichromat group also utilize more plants for fruit feeding: “vahy” (which refers jointly to vines of unknown species) and “latabariky” (*Grewia cyclea*). The difference was minor for the vahy group: only 3 additional plants for fruit (24 *vs* 21). For latabariky, which has reddish brown fruit, the difference was more pronounced (12 *vs* 6). However, group color vision phenotype was still a significant factor in feeding time per tree when latabariky was excluded (LRT: χ^2^_1_ = 6.05, *p* = 0.014).

**Figure 1:**
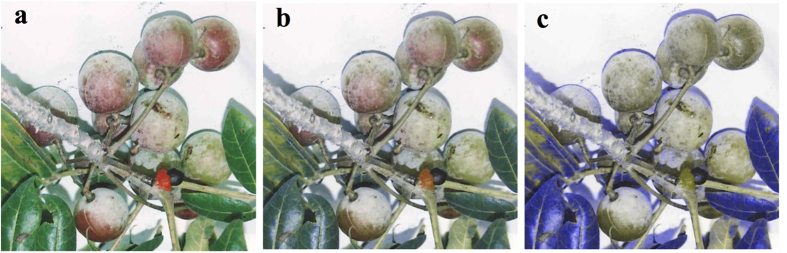
How trichromat and dichromat lemurs see the world. Harofy fruit and leaves (*Commiphora* sp.) as viewed by (**a**) a human trichromat, (**b**) a sifaka trichromat, and (**c**) a sifaka dichromat with a 558 nm M/L cone. Note that although the fruits are detectable to all color vision phenotypes, the red indicator of the degree of ripeness is visible only to individuals with trichromatic color vision. Sifaka cone sensitivities derived from *Propithecus coquereli* using electroretinogram flicker photometry^6^. Sifaka image corrections performed using Color Vision Simulator^62^. Photograph taken by R. Lewis.

**Figure 2:**
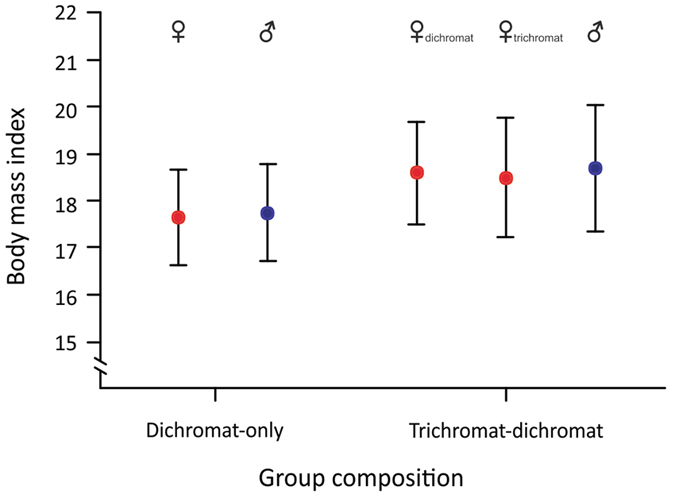
BMI by group and individual color vision phenotype. Mean and standard error for BMI predicted by the three-level color vision phenotype GLMM for dichromat males and females residing in dichromat-only groups and dichromat males, dichromat females, and trichromats residing in dichromat-trichromat groups.

For three species, the trichromat social group utilized more individual trees for fruit feeding despite using fewer individual trees for plant parts overall. Two of these three were minor differences, where the trichromat group only used one more than the next highest social group. However, for the third species, “harofy” (*Commiphora* sp.), the trichromat group used 24 more trees for fruit than the next highest social group (a 42% increase), despite using 25 fewer harofy trees (14%) than that group overall. Notably, harofy fruits are reddish upon ripening (Fig. 1), hinting at a potential trichromat group advantage.

Although our feeding data are limited in several ways (e.g., limited sampling of trichromat individuals and groups, as well as lack of data on food color properties), we nonetheless identified some of the first evidence of a potential group-level trichromat advantage for feeding on fruit. In contrast to studies of capuchins^15^ and squirrel monkeys^26^, which found no significant effects of color vision phenotype on gross-scale feeding behavior, our data suggest that both trichromat females and individuals residing in a group with trichromats spend more of their total time budget on fruit feeding than individuals in dichromat-only groups. Furthermore, during the dry season when fruit is scarce, members of the trichromat group have longer feeding bouts in fruit patches than members of dichromat-only groups. This result is consistent with hypotheses of a trichromat advantage in detecting productive food patches at a distance^12^,^52^ and supports predictions of the mutual benefit of association hypothesis^20^,^26^,^33^. Because fruit intake rates did not differ with any aspect of color vision phenotype (similar to platyrrhines refs 23,27,28), this result offers a potential mechanism to explain why members of sifaka trichromat social groups across the Ankoasifaka Research Station exhibit better body condition during the dry season.

## Conclusions

In this study, we demonstrate the existence of polymorphic trichromacy in a wild population of Verreaux’s sifaka in the Kirindy Mitea National Park and explore possible adaptive benefits of trichromacy. The results of our morphometric, demographic, and feeding behavior analyses offer some of the first evidence for a group-level benefit of associating with trichromats in wild primates. Although our findings on feeding behavior should be interpreted with caution due to the limitations of our data, these results suggest that in Verreaux’s sifaka, trichromats experience an advantage for finding more productive fruit patches during the energetically stressful dry season. This feeding advantage translates into measurable differences in male and female body condition between members of trichromat and dichromat social groups, as well as a possible reproductive benefit among trichromat females for increased infant survival to the first year. Most studies of wild platyrrhines have found no differences between trichromats and dichromats in many aspects of fruit feeding (e.g., intake rate, proportion of feeding time)^15^,^27^,^53^ or correlates of reproductive success^29^, suggesting that polymorphic trichromacy in these species may be maintained through niche differentiation^12^. Our findings suggest that selection for polymorphic trichromacy may differ across primates, particularly species living in small cohesive social groups. Consequently, sifaka and similar lemurs may offer an excellent alternative model for exploring the evolution and maintenance of polymorphic trichromacy in primates.

## Methods

### Morphometric and Demographic Data

Between 2006 and 2016, 63 sifaka in 9 social groups in and around the 1 km^2^ trail system at Ankoatsifaka Research Station (20°47′17″S, 44 ° 10′08″E) at Kirindy Mitea National Park were captured and individually marked. Four groups have been the focus of long-term research, while data on the remaining groups have been collected opportunistically for demographic, genetic, and morphometric studies. Group size during the period of 2007 to 2014 ranged between 2 to 11 individuals of all age classes (0–3 adult males, 1–3 adult females)^44^. Beginning in 2006, lemurs were sedated during annual dry season captures occurring within a 5-week period between June and July. For each captured individual, body length (crown to base of tail) and mass measurements were collected, and two small biopsy punches (2 mm each) were taken from the ear and preserved in 100% ethanol. Capture and collection protocols were performed in accordance with protocols approved by the University of Texas at Austin Institutional Animal Care and Use Committee (protocol numbers 05101801, 08110301, AUP-2011-00143, and AUP-2014-0036; see Lewis^54^ for further method details). This study complies with all established IACUC guidelines and Malagasy law. We also collected data on infant birth and survival from censuses from 2006 to 2015. Of the 63 individuals in this study, tissue samples were available for 55 individuals from 9 social groups (31 females, 24 males), body mass and length measurements for 40 mature individuals from 9 groups (13 females, 27 males), and infant birth and survival data for 14 mothers from 6 groups (Supplementary Table 1).

### M/L opsin locus genotyping

We extracted genomic DNA from tissue samples using DNeasy Blood and Tissue Kits (Qiagen). To genotype the M/L opsin polymorphism, we followed Montague^26^ and custom-designed a TaqMan SNP genotyping assay (Applied Biosystems) targeting a spectral tuning site in exon 5 (residue 285) that is known to be variable in lemurs^5^,^6^,^8^. At this site, a single SNP shifts the spectral absorbency of the M/L cone from 543 nm to 558 nm^6^. Polymerase chain reactions (PCRs) to amplify this locus were performed on a Mastercycler RealPlex (Eppendorf) with the following conditions: (1) 10 min at 95 °C; (2) 40 cycles of 15 sec at 95 °C followed by 1 min at 60 °C; (3) 2 min at 60 °C; (3) 10 sec at 55 °C; (4) 55–95 °C at 0.5 °C increments; (5) 15 sec at 95 °C. Each PCR included 10 μL TaqMan Mastermix, 0.5 μL TaqMan genotyping assay, and ~10 ng DNA template, along with HPLC-purified H_2_0 to reach a total 20 μL volume. Two replicate PCRs were performed for each individual from the same extraction. To identify positive controls and confirm TaqMan results, we also genotyped five individuals using traditional PCR and Sanger sequencing (conditions as in ref. 8).

### Feeding behavior data

Behavioral data were available for 28 adult individuals from four social groups (Supplementary Table 1). Between October 2007 and June 2015, we recorded individual feeding behavior (feeding bout duration, plant part consumed, morphospecies, plant identification number) continuously during half- or near-full day focal animal follows^55^ (*N* = 3137 hrs, mean ± standard deviation: 112 ± 83 hours/individual), representing 20,080 total feeding bouts (628 ± 581 feeding bouts/individual). We defined bouts as continuously feeding on a plant part, and a new bout was recorded following breaks in processing or chewing greater than five seconds. We defined ‘tree feeding bout’ as the sum of all individual bouts by a focal animal in each feeding tree for a particular plant part before moving to a new tree. We categorized plant parts as either fruit, buds, flowers, seeds, mature leaves, young leaves, bark, vines, or stems. Beginning in 2013, we also recorded feeding intake rates (bites/min) when visibility and bout length permitted through one-minute continuous sampling. Because this dataset was not directly collected to address questions regarding color vision, we did not record the color of food items or assess fruit ripeness. Our data do, however, differentiate between seed predation (when the fruit is discarded and only seed is consumed) and frugivory. The observers were blind to the color vision phenotypes of the focal animals.

### Statistical analyses

To examine the effects of color vision phenotype on body condition, reproduction, and feeding behavior, we fitted LMMs and GLMMs using the lme4^56^ and lmerTest^57^ packages in R version 3.2.3^58^. For each individual, color vision phenotype was categorized as (1) ‘individual color vision phenotype’ (i.e., dichromat or trichromat) based on whether the individual was homozygous or heterozygous at the *M*/*LWS* opsin exon 5 locus, and (2) ‘group color vision phenotype’ based on whether the individual was a member of a social group containing any trichromats. If group phenotype was significant, we also categorized individuals by a three level color vision factor (‘dichromat in a dichromat-only group’, ‘dichromat in a trichromat group’, and ‘trichromat’) in a *post-hoc* analysis to separately assess the contributions of dichromats and trichromats to any significant differences for trichromat groups.

#### Body condition and reproduction

We examined the effects of color vision phenotype on three presumed correlates of reproductive success: body mass index (BMI), reproductive output, and infant survival to the first year. We calculated BMI for each adult based on mass and crown-tail length collected during annual dry season captures as mass (kg)/length (m)^2^. Because of the longitudinal nature of the study, some individuals were measured in multiple years. We performed LMMs including sex, pregnancy status (based on whether a female gave birth later that year), and either individual or group color vision phenotype as main effects. These models included three random effects to account for other sources of variation in BMI: the year of measurement, individual identity (controlling for repeated measurements), and social group as crossed random effects (*vs*. nested) because some males transferred between study groups over time. For this and all analyses, we used likelihood ratio tests (LRTs) to compare the fit of the color vision phenotype models to a null model excluding the color vision phenotype.

To assess effects of color vision phenotype (individual or group) on reproduction, we used census data for all available infants to calculate two binary reproductive measures: reproductive output (i.e., for each year a female was observed, did she give birth: yes/no), and infant survival through the first year. Infant survival to the first year has previously been used as a measure of successful reproduction in Verreaux’s sifaka^49^. Because both measures were binary, we fitted binomial GLMMs with a log-link function, and separately tested the main effects of individual phenotype and group phenotype. All models included random effects of mother’s identity (controlling for repeated measuring and differences in phenotypic quality refs 29 and 59) nested within social group, and the year the measurement was taken or infant was born.

#### Feeding behavior

We used LMMs and GLMMs to explore the relationship between color vision phenotype and three aspects of feeding behavior: monthly feeding time budget, intake rate, and bout length per feeding tree. Two males in the feeding behavior dataset moved between groups during the course of the study, spending one or two focal months with one group, and ten focal months with a second group. Due to statistical concerns, we restricted the data from these males to only the groups in which they resided for the longest time period. Because of strong seasonal variation in diet^42^,^43^, we excluded data from inter-season transitional months (Apr, Dec) and either included season (wet/dry) as a fixed effect in the models or analyzed wet and dry seasons separately. We also included sex as a fixed effect in all models.

For monthly time budget analyses, we calculated the total time spent feeding, the time spent feeding on fruit, and the time spent feeding on young leaves as proportions of total observation time per month for each individual. The number of sampled days varied by individual (range = 1–4 days/month, mean = 1.3 days). For these analyses, we excluded focal months where the target item was not consumed and then logit-transformed the proportion data^60^. We performed LMMs on the logit-transformed proportions with fixed effects of sex, season, and either individual or group color vision phenotype, and random effects of focal individual (due to potential variation in preferences) nested within social group (due to potential group-level differences in preference and home range quality ref. 25), the year data were collected (due to inter-annual variation in severity of seasonality), and the month data were collected. To account for multiple testing of proportion data, we adjusted p-values using the Benjamini-Hochberg method^61^ in *R*. For fruit intake rate analyses, we performed GLMMs with a Poisson distribution for fixed effects of sex, season, and either individual or group color vision phenotype, with random effects of focal individual identity nested within social group, month and year of data collection, and tree morphospecies. For tree feeding bout lengths, we performed LMMs on the log-transformed bout length with fixed effects of sex and either individual or group color vision phenotype, with random effects of focal individual identity nested within social group, month and year of data collection, tree morphospecies, and observer identity. Wet and dry season data were analyzed separately.

## Additional Information

**How to cite this article**: Veilleux, C. C. *et al*. Group benefit associated with polymorphic trichromacy in a Malagasy primate (*Propithecus verreauxi*). *Sci. Rep.* **6**, 38418; doi: 10.1038/srep38418 (2016).

**Publisher's note:** Springer Nature remains neutral with regard to jurisdictional claims in published maps and institutional affiliations.

## Supplementary Material

Supplementary Material

## Figures and Tables

**Table 1:** Model parameters for effects of color vision phenotypes, sex, and season on the proportion of time sifaka spend feeding on fruit.

Model	Fixed Effects	Estimate ± S.E.	df^1^	t value	*p*
individual	(Intercept)	−3.95 ± 0.38	13.48	−10.27	<0.001
	**Individual: trichromat**	**0**.**54** ± **0**.**23**	**227**.**69**	**2**.**34**	**0**.**020**
	Sex: male	0.12 ± 0.18	227.52	0.71	0.476
	**Season: wet**	**1**.**00** ± **0**.**43**	**7**.**94**	**2**.**31**	**0**.**0499**
group	(Intercept)	−3.94 ± 0.38	13.34	−10.37	<0.001
	**Group: trichromat**	**0**.**52** ± **0**.**17**	**229**.**03**	**2**.**98**	**0**.**003**
	Sex: male	−0.06 ± 0.16	227.97	−0.37	0.711
	**Season: wet**	**1**.**00** ± **0**.**43**	**8**.**04**	**2**.**32**	**0**.**049**
3-level	(Intercept)	−3.95 ± 0.38	13.63	−10.31	<0.001
	3-level color vision phenotype				
	*trichromat group mates*	*0*.*47* ± *0*.*26*	*227*.*32*	*1*.*84*	*0*.*068*
	**trichromats**	**0**.**55** ± **0**.**23**	**226**.**76**	**2**.**38**	**0**.**018**
	Sex: male	−0.03 ± 0.19	226.93	−0.18	0.858
	**Season: wet**	**1**.**00** ± **0**.**43**	**8**.**03**	**2**.**31**	**0**.**05**

Response variable is logit-transformed. Reference categories: dichromats (for Individual), dichromat-only group (for Group), and dichromats in dichromat-only group (for 3-level phenotype), females (for Sex), and dry season (for Season). Random effects: focal ID/group ID, month, and year. Significant effects are shown in bold, trends (0.05 < *p* < 0.10) are italicized.

^1^lmerTest package t-tests use Satterthwaite approximations to df to calculate p-value.

**Table 2:** Model parameters for effects of color vision phenotypes and sex on fruit feeding tree bout length in sifaka.

Season	Model	Fixed Effects	Estimate ± S.E.	df^1^	t value	*p*
wet season	individual	(Intercept)	5.69 ± 0.49	1.2	11.67	0.036
(*n* = 501 bouts)		Individual: trichromat	0.36 ± 0.23	11.0	1.56	0.147
		Sex: male	0.12 ± 0.15	12.3	0.78	0.450
	group	(Intercept)	5.76 ± 0.48		11.993	
		Group: trichromat	0.09 ± 0.16		0.556	
		Sex: male	0.03 ± 0.15		0.184	
dry season	individual	(Intercept)	5.63 ± 0.33	1.0	17.12	0.034
(*n* = 440 bouts)		Individual: trichromat	0.12 ± 0.15	9.0	0.81	0.439
		Sex: male	0.10 ± 0.10	14.5	0.96	0.355
	group	(Intercept)	5.62 ± 0.34	1.00	16.61	0.037
		Group: trichromat	**0**.**24** ± **0**.**10**	**428**.**7**	**2**.**33**	**0**.**021**
		Sex: male	0.05 ± 0.09	424.8	0.53	0.596

Response variable is log-transformed. Reference categories: dichromats (for Individual), dichromat-only group (for Group), females (for Sex). Random effects: focal ID/group ID, observer ID, tree morphospecies, month, and year. Significant effects are shown in bold.

^1^lmerTest package t-tests use Satterthwaite approximations to df to calculate p-value.
