# Supplementary material for: Group benefit associated with polymorphic trichromacy in a Malagasy primate (Propithecus verreauxi)
Source: Sci Rep. 2016 Dec 2;6:38418. doi: 10.1038/srep38418 (PMC5133583; doi:10.1038/srep38418)
Supplement: Supplementary Material [file srep38418-s1.pdf]

Group benefit associated with polymorphic trichromacy in a Malagasy primate (*Propithecus verreauxi*)

Carrie C. Veilleux[1]*, Clara J. Scarry[1], Anthony Di Fiore[1], E. Christopher Kirk[1], Deborah A. Bolnick[1,2], and Rebecca J. Lewis[1]

[1]Department of Anthropology, University of Texas at Austin, 2201 Speedway Stop C3200, Austin, TX, 78712

[2]Population Research Center, University of Texas at Austin, Austin, TX, 78712.

*Corresponding author: carrie.veilleux@utexas.edu

Supplementary Material

Suppl. Table 1. Summary of data available for groups and individuals sampled in this study

Suppl. Table 2. Number of focal hours and individuals used in feeding behavior analyses

Suppl. Table 3. Parameters for LMMs of effects on proportion of total time spent feeding and time spent feeding on young leaves

Suppl. Table 4. Parameters for LMMs of effects on sifaka intake rate for fruit

Suppl. Figure 1. Preliminary comparison of habitat characteristics between trichromat and dichromat groups

Supplementary Table 1. Summary of data available for groups and individuals sampled in this study. Data abbreviations: body mass index (BMI), reproduction (R), feeding behavior (FB), color vision genotyping (G), adult (A), subadult (S). For 8 males, color vision genotyping was not available. However, as males, they are phenotypically dichromat (D*). Italicized lines represent males that change groups during the study period, and so are listed in two groups. While feeding data are available for Group VI, there were too few observation hours to include in statistical analyses (36 total hours across 4 individuals, *vs* 700 to 830 hrs for Groups II, III, IV, and V).

Group	ID	Data	Sex	Color Vision	BMI Years	R Years	FB Years
I	Cliff	BMI, G	M	D	2007 (A)		
	Colbert	G	M	D			
	Juliet	BMI, R, G	F	D	(A): 2007,2010,2011,2013	2006-14	
	Khaleb	BMI	M	D*	2014 (A)		
	Louise	BMI, R, G	F	T	2011,2015 (A)	2010-14	
	Meyers	BMI, G	M	D	2011 (A)		
	Micka	BMI, G	M	D	2008,2010 (A)		
II	Achilles	BMI,FB, G	M	D	2011-12 (S)		2011
	Kida	R, G	F	D		2008	
	Bevo	BMI, G	M	D	2006 (A)		
	Joker	BMI, FB, G	M	D	2013 (A)		2011-14
	Lascaux	BMI, G	M	D	2011 (S)		
	Omby	BMI, FB, G	M	D	(A): 2007-08, 2010-11		2008, 2010-11
	Robert	*BMI, FB, G*	*M*	*D*	*2008 (A)*		*2008*
	Savannah	BMI, FB, R, G	F	T	(A): 2007-08, 2010, 2012	2006-14	2007-08, 2010-15
	Sakay	G	M	D			
	Stacey	G	F	T			
	William	BMI, FB	M	D*	2012 (A)		2011, 2013-15
	Zebu	*BMI, G*	*M*	*D*	*2007 (A)*		
	Zafiry	G	F	D			
	Zebra	G	F	T			

Group	ID	Data	Sex	Color Vision	BMI Years	R Years	FB Years
	Zena	BMI, FB, R, G	F	T	2007 (S), (A): 2010-11, 2013, 2015	2008-14	2008, 2010-15
III	Glen	BMI, FB, G	M	D	(A): 2006-08, 2010, 2012		2007-08, 2010-11
	Albert	FB	M	D*			2013-14
	Hester	BMI, FB, R, G	F	D	(A): 2010, 2012	2007-14	2007-08, 2010-14
	Harietta	G	F	D			
	Hope	G	F	D			
	Isaac	*BMI, FB*	*M*	*D**	*2015 (A)*		*2013-15*
	Quincy	BMI, G	M	D	2006-07 (A)		
	Thor	BMI, FB	M	D*	2013 (A)		2013-15
	Vanilla	BMI, FB, R, G	F	D	(A): 2006-08, 2010-13, 2015	2006-14	2007-08, 2010-15
	Vary	FB, G	M	D			2011
	Velo	FB, G	F	D			2014-15
IV	Baobab	BMI, G	M	D	2006 (A)		
	Dalton	BMI, FB, G	M	D	2007 (A)		2007
	Fantasia	BMI, FB, R, G	F	D	2006-08 (A)	2006-09	2007-08
	Isaac	*FB*	*M*	*D**			*2011*
	Marsu	BMI, FB	M	D*	2012 (A)		2011, 2013-15
	Petunia	BMI, FB, R, G	F	D	(A): 2006-07, 2011-12	2006-12	2007-08, 2010-11
	Papay	G	F	D			
	Prisca	G	F	D			
	Pamella	G	F	D			
	Frog	G	F	D			
	Manga	G	F	D			
	Azalea	G	F	D			
	Kamilla	G	F	D			

Group	ID	Data	Sex	Color Vision	BMI Years	R Years	FB Years
	Quincy	BMI, FB, G	M	D	(A) 2008, 2010-11		2008, 2010-11
	Robert	*BMI, G*	*M*	*D*	*2007 (A)*		
	Rija	FB	M	D*			2014
	Rose	BMI, FB, R, G	F	D	(A): 2010, 2012, 2014	2007-13	2007-08, 2010-15
	Titan	*BMI, FB, G*	*M*	*D*	*2010 (A)*		*2008, 2010-11*
	Zebu	*BMI, G*	*M*	*D*	*2006 (S)*		
V	Abby	BMI, FB, R, G	F	D	(A): 2007-08, 2010-12, 2014	2007-13	2007-08, 2010-15
	Asterix	G	F	D			
	April	FB, G	F	*D*			2014-15
	Ana	FB, R, G	F	*D*		2013-14	2013-15
	Sherlock	BMI, FB, G	M	D	(A): 2012, 2015		2010-11, 2013-15
	Xavier	BMI, FB, G	M	D	(A): 2007-08, 2010-11		2007-08, 2010-11
	Titan	*FB, G*	*M*	*D*			*2007*
VI	Emily	BMI, FB, R, G	F	D	(A): 2008, 2012, 2014-15	2013-14	2008, 2014-15
	Nancy	BMI, FB, R, G	F	T	(A): 2008, 2015	2013-14	2014
	Doughnut	FB, G	M	D			2008
	Orion	BMI	M	D*	2014 (A)		
	Rich	BMI, FB, G	M	D	2008 (A)		2015
VII	Obama	BMI, G	M	D	2008 (A)		
VIII	Belina	BMI, G	F	T	2008 (A)		
	Xerxes	BMI,G	M	D	2008 (A)		
IX	Tilda	G	F	D			
**Total N**	63			55	40	14	32 (28 excluding GrVI)

Supplementary Table 2. Number of focal hours and individuals used in feeding behavior analyses for each social group by sex and individual color vision phenotype.

	Females			
**Group**	**Trichromat**	**Dichromat**	**Males**	**Total**
II	*N* = 453.1 hrs (2)	-	*N* = 375.9 hrs (5)	*N* = 829 hrs (7)
III	-	*N* = 494.1 hrs (3)	*N* = 331.2 hrs (5)	*N* = 825.3 hrs (8)
IV	-	*N* = 481.2 hrs (3)	*N* = 327.7 hrs (6)	*N* = 808.9 hrs (9)
V	-	*N* = 415.2 hrs (3)	*N* = 285.2 hrs (3)	*N* = 700.4 hrs (6)

Number of individuals for each phenotype listed in parantheses.

Supplementary Table 3. Parameters for LMMs of effects of color vision phenotypes, season, and sex on proportion of time spent feeding overall and proportion of time spent feeding on young leaves.

Feeding Measure	Model	Fixed Effects	Estimate ± S.E.	df[1]	t value	*p*
total feeding time	individual	(Intercept)	-0.45 ± 0.16	7.7	-2.84	0.022
406 focal months		Individual: trichromat	0.09 ± 0.07	390.5	1.28	0.201
		**Sex: male**	**-0.15 ± 0.05**	**389.6**	**-2.90**	**0.004**
		Season: wet	-0.13 ± 0.11	8.7	-1.25	0.245
	group	(Intercept)	-0.44 ± 0.16	7.7	-2.75	0.026
		Group: trichromat	0.02 ± 0.05	15.3	0.38	0.710
		**Sex: male**	**-0.18 ± 0.05**	**20.3**	**-3.56**	**0.002**
		Season: wet	-0.13 ± 0.11	8.7	-1.21	0.258
young leaf feeding	individual	(Intercept)	-3.36 ± 0.38	14.6	-8.76	<0.001
321 focal months		Individual: trichromat	-0.07 ± 0.19	7.4	-0.37	0.724
		Sex: male	-0.23 ± 0.14	16.9	-1.68	0.111
		**Season: wet**	**1.37 ± 0.55**	**8.2**	**2.49**	**0.037**
	group	(Intercept)	-3.37 ± 0.38	14.4	-8.84	<0.001
		Group: trichromat	-0.02 ± 0.14	13.2	-0.16	0.874
		Sex: male	-0.21 ± 0.13	16.4	-1.68	0.112
		**Season: wet**	**1.37 ± 0.55**	**8.2**	**2.48**	**0.037**

Response variables are logit-transformed. Reference categories: dichromats (for Individual), dichromat-only group (for Group), females (for Sex), and dry season (for Season). Random effects: focal ID/group ID, month, and year. Significant effects are shown in bold.
[1]lmerTest package t-tests use Satterthwaite approximations to df to calculate p-value.

Supplementary Table 4. Parameters for generalized LMM of the effects of color vision phenotypes, sex, and season on sifaka intake rate (bites/min) for fruit.

**Model**	**Fixed Effects**	**Estimate ± S.E.**	**z value**	***p***
individual	(Intercept)	2.09 ± 0.13	16.04	<0.0001
	Individual: trichromat	-0.06 ± 0.08	-0.85	0.397
	Sex: male	-0.03 ± 0.05	-0.61	0.541
	Season: wet	0.07 ± 0.19	0.39	0.699
group	(Intercept)	2.09 ± 0.13	16.21	<0.0001
	Group: trichromat	-0.05 ± 0.06	-0.88	0.378
	Sex: male	-0.01 ± 0.04	-0.34	0.736
	Season: wet	0.08 ± 0.19	0.43	0.669

Reference categories: dichromats (for Individual), dichromat-only group (for Group), females (for Sex), and dry season (for Season). Random effects: focal ID/group ID, month, and year.

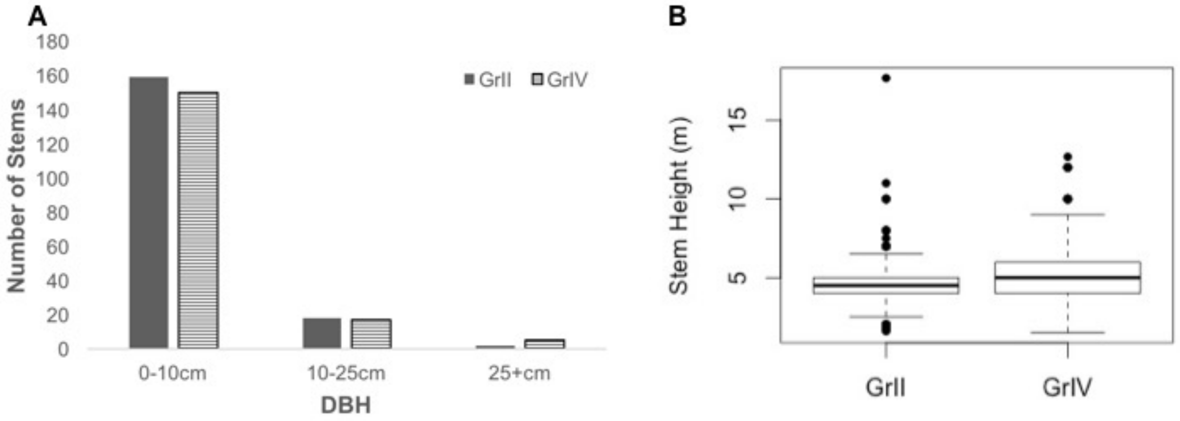

Supplemental Figure 1. Preliminary comparison of habitat characteristics between trichromat and dichromat groups. Stem density and height for three 50 m x 2 m transects were each available for one trichromat group (Group II) and one dichromat group (Group IV) at KMNP. Neither stem density (Chi-square test: $\chi^2_2 = 1.44$, p = 0.487) or stem height (Wilcoxon rank sum: W = 15998, $p = 0.249$) differed significantly between groups. Data: CC Veilleux, unpublished data.
